# Authors’ Response to Peer Reviews of “Sexual Health Assessment Is Vital to Whole Health Models of Care”

**DOI:** 10.2196/40159

**Published:** 2022-07-28

**Authors:** Alex Uzdavines, Drew A Helmer, Juliette F Spelman, Kristin M Mattocks, Amanda M Johnson, John F Chardos, Kristine E Lynch, Michael R Kauth

**Affiliations:** 1 South Central Mental Illness Research, Education, and Clinical Center Michael E. DeBakey VA Medical Center Houston, TX United States; 2 Center for Innovations in Quality, Effectiveness, and Safety Michael E. DeBakey VA Medical Center Houston, TX United States; 3 Department of Medicine Baylor College of Medicine Houston, TX United States; 4 VA Connecticut Healthcare System West Haven, CT United States; 5 Yale School of Medicine New Haven, CT United States; 6 VA Central Western Massachusetts Healthcare System Leeds, MA United States; 7 University of Massachusetts Medical School Worcester, MA United States; 8 VA Cheyenne Medical Center Cheyenne, WY United States; 9 VA Palo Alto Health Care System Palo Alto, CA United States; 10 Stanford University Palo Alto, CA United States; 11 VA Informatics and Computing Infrastructure VA Salt Lake City Health Care System Salt Lake City, UT United States; 12 Department of Internal Medicine University of Utah School of Medicine Salt Lake City, UT United States; 13 LGBTQ+ Health Program Patient Care Services Veterans Health Administration Washington, DC United States

**Keywords:** sexual health, sexual health assessment, veteran, health equity, health assessment, whole health model, communication, communication barrier, technological barrier, health care, sexuality, sexual orientation, gender identity, sex, gender, model, care, barrier, well-being, comfort, assessment, EHR, electronic health record, quality, equity

*This is the authors’ response to peer-review reports for “Sexual Health Assessment Is Vital to Whole Health Models of Care.”*

## Round 1 Review

### Reviewer BM [1]

Comment: This is an excellent paper [2] that should be published. It’s well-written, informative, and important to disseminate to the health care community. This paper addresses an initiative by the Veterans Affairs (VA) health care system to address an issue important for providing high-quality comprehensive patient care. It is great that addressing sexual health assessment in their electronic health record (EHR) is being implemented by the VA since federal programs often influence national implementation. The additions presented in this paper need to be incorporated into other EHR programs, like Epic. Integration of detailed sexual health information in patient documentation is important to address preventive care, promote healthy sexual functioning, and optimize overall health and well-being. Having done research on the use of surveys to address sensitive topics such as sexual health and sexually transmitted infection risk, I found that patients of all ages are very willing to answer honestly (and in detail) about their sexual health on surveys, which then provides a time-efficient and useful way to open this discussion. This approach also allows health care providers to introduce these sensitive topics and, when patients are willing, provide health education, health promotion, and appropriate treatment when needed. I have also used this approach in my own clinical practice. This use of surveys to obtain and open the discussion on sensitive health issues has been documented in numerous research projects. It has also been shown to promote patient satisfaction with their care since it increases their sense of “being heard” by their providers. It also allows providers to provide more accurate care.

This paper is important to disseminate information about how a major health system has recognized the importance of sexual health assessment and found a way to implement this and incorporate it into their EHR. It also highlights the need to educate providers to orient them to the new system and provides ways to do this to assist them in better approaching sensitive health issues.

Response: Thank you for the compliments on the paper! We agree that the US federal government plays an important role in driving changes in health care delivery and that systems outside the Veterans Health Administration (VHA) will integrate sexual health into their EHRs. We also appreciate and agree with your perspective on using survey approaches to collect screening information about sensitive topics.

#### Specific Comments

Comment: Great information about the VA’s approach. A particular strength of this paper is the presentation of prompts at different levels of assessment for patients who are hospitalized and more comprehensive visits.

##### Major Comments

Comment 1: Excellent background information

Comment 2: The pocket card format is excellent and serves several purposes: makes it easy for providers to follow a template and validates the importance of this information in providing patient care, and the repetition of obtaining this data in practice with all patients reinforces the importance of obtaining this data and can reduce provider resistance to asking these questions.

Comment 3: Following the pocket card questions (comprehensive form) nicely addresses and includes the patient’s partner information in the history. This reinforces for the provider the benefit of obtaining this information. The fact that the provider/EHR asks for partner information also highlights for the patient the importance to consider their partner(s) when addressing their own sexual health. This can also be a great trigger for patient education, which moves beyond the basic “plumbing” aspect of sexual health information (eg, how things work).

Response: Thank you! Yes, we’re hopeful these kinds of educational materials will help providers navigate sexual health assessments more easily.

##### Minor Comments

Comment 4: A minor suggestion would be to provide a little more detail about provider education in the implementation of this new format. This could include role-playing with debriefing to help providers address their own concerns/reluctance to talk about sexual issues with their patients.

Response: Thanks for the suggestion! We added more detail about provider education, including the use of role-plays and practice assessments:

- Page 13, paragraph 2: “Two trainings on gender identity—one for current EHR users and one for Cerner Millennium users—have already been released. Separate trainings on sexual orientation and on sexual health (for current EHR users and for Cerner Millennium users) are nearing completion.”
- Page 14, paragraph 1: “Brief role-plays during pre-clinic stand-ups could help provider teams become comfortable using these tools.”

### Anonymous [3]

#### General Comments

Comment: Thank you for the opportunity to review this manuscript entitled: Sexual Health Assessment as Part of a Whole Health Model to Care: Improving Communication and Technological Barriers. This review paper aims to summarize barriers to assessing sexual health and to suggest some ways to overcome them. The paper summarizes sexual health issues from a new perspective (whole health model), trying to draw some practical new methods. However, several significant questions should be explained before publication.

Response: Thank you for reviewing the manuscript and the feedback!

#### Specific Comments

##### 1. Abstract

Comment: This seems to be a review article in my understanding.

Response: This is a commentary paper; we reviewed pertinent literature, but the intent is to make an argument for assessing sexual health rather than aggregate and report information and themes from prior work. We revised the title to make this clear for readers: “Commentary: Sexual health assessment is vital to whole health models of care”

Comment: I suggest reorganizing the abstract. For review articles, the abstract specifies the topic of the review and the main conclusions drawn.

Response: We revised the abstract to highlight our central argument: “If health systems, including the U.S. Department of Veterans Affairs Veterans Health Administration (VHA), incorporate sexual health into whole health models of care they could enhance preventive care, promote healthy sexual functioning, and optimize overall health and well-being.”

Comment: Additionally, why is there a holistic health model in the title and no mention of it in the abstract?

Response: We revised the abstract to clarify the link with whole health models of care: “Healthcare systems adopting whole health models of care will need to incorporate holistic assessment of sexual health...into whole health models of care they...”

Comment: Also, why the VHA is specifically mentioned needs to be answered in the abstract.

Response: We named the VHA earlier in the abstract to make readers aware the VHA would be discussed in more depth later in the abstract: “If health systems, including but not limited to the Veterans Health Administration (VHA), incorporate...”

Later in the abstract, we discuss the VHA’s changes to its EHR; we clarified that this example could be helpful for providers and administrators outside the VHA: “These examples may be helpful for other healthcare systems interested in moving to a whole health model of care.”

Comment: Finally, the purpose of the review needs to be reclarified in the abstract. As of the current version, the purpose of this paper seems to be reported too vaguely. Is the authors’ main intent to provide recommendations for development and implementation for the VHA through this review or is the target population just veterans?

Response: In addition to the specific revisions specified above, throughout the abstract we clarified that these issues are pertinent beyond the VHA:

“These examples may be helpful for other healthcare systems interested in moving to a whole health model of care”

“These interventions will need to be targeted to both providers and patients in all healthcare systems wanting to transition into whole health models of care, not just the VHA”

“Healthcare systems (i.e., both the VHA and other systems)...”

##### 2. Introduction

Comment: It is recommended to reorganize in a logical order. Similar to the summary, readers are confused by the sudden mention of veterans.

Response: We added a discussion of veterans and, for clarity, specified that we are not just discussing veterans throughout the Introduction:

“Most people, not just Veterans (Veterans are individuals who have served in the Armed Forces, regardless of combat exposure, and who are no longer on active duty after receiving an honorable discharge from military service), are sexually active and value sexuality”

“Veterans, especially women and sexual and gender minority veterans, experience a high rate of disruptions in healthy sexual intimacy. This is due to premilitary trauma, high rates of military-related injuries, multiple and often comorbid chronic illnesses (e.g., vascular disease, obesity, depression, posttraumatic stress symptoms, substance use disorders, and tobacco use), and medication side-effects that interfere with sexual desire and functioning (5,7–11). While Veterans may be exposed to risk factors that disrupt sexual health more frequently, many of these risk factors contribute to sexual dysfunction in the general population as well”

“...overlooked in clinical practice, both in the Veterans Health Administration and other healthcare systems...”

Comment: Since the first paragraph throws out the concept of sexual health. I propose to consolidate the second and third paragraphs while reducing the content. First, mention the fact that sexual health is now an integral part of overall health, then introduce the benefits of achieving sexual health (the original two or three paragraphs), and finally the current obstacles to achieving sexual health (original fourth paragraph).

Response: Thank you for feedback about the flow of the introduction. We chose to use subheadings to highlight changes in discussion from benefits of and risks to sexual health to incorporating sexual health (and its assessment) in whole health models.

##### 3. Barriers to Assessing Sexual Health

Comment: I personally recommend independent secondary headings.

Response: While we did use subheadings to highlight changes in direction in the original draft, we changed the formatting of the subheadings to better showcase them for readers and to conform to JMIR’s formatting requirements.

Comment: Of the three obstacles mentioned in the paper, the first is too long to describe. I suggest points 2 and 3 need to be longer to make the structure of the paper smoother.

Response: We acknowledge that we spend more time discussing the communication gap caused by providers’ incorrect beliefs about patients’ willingness to discuss sexual health. This is largely because this topic is more novel and less discussed than technological barriers encountered in EHRs. In addition, these technological barriers are already well on the way to being overcome whereas the patient-provider communication gap is more nuanced.

##### 4. Benefits of Assessing Sexual Health

Comment: I personally recommend independent secondary headings here also.

Response: Thank you for this suggestion! We added additional headings throughout the paper.

Comment: Assessing the benefits of sexual health highlights the importance of sexual health, which is the point of the entire review. I think this paragraph should be moved to the front.

Response: We agreed about the structural issue and moved this section to immediately follow the Introduction.

##### 5. Overcoming Barriers to Sexual Health Assessment

Comment: I think patient education is a key point in achieving sexual health assessment and needs to be covered in detail.

Response: We discuss patient education in the subsection “Patient Education.” We reformatted the subheading to highlight this subsection and conform with JMIR’s formatting requirements. In addition, we added the following update about VHA’s plans for patient education:

- Page 19, paragraph 2: “To assist with this, the VHA is developing a patient-facing factsheet about SOGI that will be posted to the VA’s public website during Pride Month (June) 2022.”

Additionally, the core of our argument from the Barriers section is that providers need to be the ones to take charge in assessing patients’ sexual health. Based on the evidence we review in that section, providers’ unwillingness to query about sexual health, because they believe patients would be offended, is a far larger barrier than patient-side communication barriers. That’s not to say patient education isn’t important. We believe (and argue that) provider education is more important for this topic.

To clarify this issue, we added this paragraph to the end of the Patient Education section:

- Page 14, paragraph 2: “The evidence we reviewed above points to the gap in patient-provider communication about sexual health largely due to provider perception. This appears to be driven by providers’ beliefs that patients are not willing to discuss their sexual health when, in fact, they are. While veteran educational interventions are important, provider education is critical for promoting sexual health assessment.”

##### 6. Conclusion

Comment: I think the conclusion of the current version needs to be reorganized. In short, if it is too long it leads to a loss of readability. The conclusion needs to be concise and outline what the paper does exactly, such as what problems were found and what solutions were proposed.

Response: Thank you for the feedback about readability. We condensed the summary paragraphs and information about pre-exposure prophylaxis that may have contributed to the lack of clarity. The first paragraph of the Conclusion now reads:

- Page 14, paragraph 2: “While sexual health is an important part of overall health, health care providers do not routinely assess patients’ sexual health. The primary barriers seem to be providers’ beliefs that patients will be offended if asked about sexual health and logistical barriers to assessing sexual health. We believe that adding sexual health assessments directly to EHRs and pairing these changes with provider education about using these tools will help. Focusing provider education on addressing the belief gap between providers and patients (i.e., sharing evidence that patients are largely comfortable with sexual health assessment) and interventions to increase providers’ comfort with discussing sexual health will likely increase the impact of logistical changes. In addition, focusing on patients’ priorities and sexual well-being could potentially increase patient engagement in care, and enhance the whole health of our patients.”

### Anonymous [4]

#### General Comments

Comment: This paper’s [1] idea is new, but unfortunately, there is no structured format for the abstract or paper. The study methods are not specified, and the conclusion is so short and insufficient.

#### Specific Comments

##### Major Comments

Comment 1: The study needs a structured format.

Comment 2: Please specify different parts of the study such as the methods and results.

Response: We believe there was a misunderstanding about this paper. It is a commentary not an original research study. There are no structured methods or results sections because there were no empirical methods or results. We hope that clarifying this in the title and providing additional structure in the abstract will prevent a similar misunderstanding for future readers.

Comment 3: Are technological barriers just related to the lack of structured data fields for this information within its current EHR or note titles?

Response: These are the primary technological barriers discussed in the literature and that we experience as health care providers and researchers, yes. “Lack of structured data fields” is also shorthand for a great deal of informatics and data architecture decision-making that is beyond the scope of this manuscript.

## Abbreviations

EHR: electronic health record
VA: Veteran Affairs
VHA: Veterans Health Administration

## References

[1] Darling-Fisher C (2022). Peer review of "Sexual Health Assessment Is Vital to Whole Health Models of Care". JMIRx Med.

[2] Uzdavines A, Helmer DA, Spelman JF, Mattocks KM, Johnson AM, Chardos JF, Lynch KE, Kauth MR (2022). Sexual health assessment is vital to whole health models of care. JMIRx Med.

[3] Anonymous (2022). Peer review of "Sexual Health Assessment Is Vital to Whole Health Models of Care". JMIRx Med.

[4] Anonymous (2022). Peer review of "Sexual Health Assessment Is Vital to Whole Health Models of Care". JMIRx Med.

